# Expression of microRNA induced by postoperative delirium‐like behavior is associated with long‐term default mode network disruption: Sequencing and a secondary analysis of resting‐state fMRI data

**DOI:** 10.1111/cns.70038

**Published:** 2024-09-24

**Authors:** Yang Liu, Huiru Feng, Huiqun Fu, Binbin Nie, Tianlong Wang

**Affiliations:** ^1^ Department of Anesthesiology Xuanwu Hospital, Capital Medical University Beijing China; ^2^ National Clinical Research Center for Geriatric Diseases Beijing China; ^3^ Institute of High Energy Physics Chinese Academy of Sciences Beijing China

**Keywords:** default mode network, microRNA, neuroinflammation, postoperative delirium, resting state functional magnetic resonance imaging

## Abstract

**Aims:**

Resting state functional magnetic resonance imaging (rs‐fMRI) has been widely used in studying default mode network (DMN) changes in postoperative delirium (POD). Reproducibility and interpretability of the analyzing results remain insufficiently studied.

**Methods:**

Delirium‐like behavior was induced by tibial fixation surgery under isoflurane anesthesia. Firstly, we evaluated delirium‐like behavior and inflammatory responses in hippocampus and systemic level. Then the expressions of microRNA (miRNA) and target gene were sequenced and validated. Afterwards the functional connectivity (FC) in DMN was analyzed. Finally, results were correlated with DMN changes.

**Results:**

POD‐like behavior caused significant changes of miR‐34b‐5p, miR‐328‐5p, and miR‐3505 in miRNA level and *Nos1*, *Tubb3*, and *Gys1* in the gene level. The FC in left and right hippocampus (L‐Hip and R‐Hip) and right auditory cortex (R‐AC) was found significantly changed. Significant correlations were found in FC_L‐Hip/R‐AC_ and FC_R‐Hip/R‐AC_ for miR‐34b‐5p and miR‐3505, as well as *Nos1* and *Tubb3*. For miR‐328‐5p, no significant correlations were found.

**Conclusion:**

Our study demonstrates that POD‐like behavior induced significant miRNA and gene expression changes were associated with hippocampus related long‐term FC disruption in DMN. The results increased reproducibility and interpretability for standardized rs‐fMRI data analysis, as well as providing potential targets for postoperative delirium treatment.

## Graphical abstract

In this research we focused on the objective changes behind default mode network disruption induced by delirium‐like behavior in aged rat induced by isoflurane anesthesia and tibia fracture fixation surgery. The study not only pointed out long‐term changes in miRNA and target gene, but also improves interpretability and reproducibility of resting state functional magnetic resonance imaging data analysis.

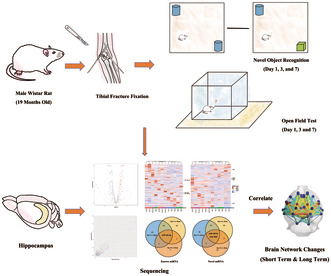

## 1 INTRODUCTION

Postoperative neurocognitive disorders (PNDs) is a umbrella term which mainly includes emergence excitation/delirium, postoperative delirium (POD), delayed neurocognitive recovery (dNCR), and postoperative mild/major neurocognitive disorder (POCD).^1^ As one of the most commonly seen PNDs subtype in orthopedic surgery (for up to 50%–70% in high risk patients),^2^ POD has been proved to increase the duration of hospital stay, morbidity and mortality,^3^ and the most significant of which, highly correlated with long‐term cognitive decline.^4^, ^5^ In this case, understanding the mechanism and development of POD is of great significance in both clinical practice and preclinical experiments.

In recent years, the use of resting state functional magnetic resonance imaging (rs‐fMRI) has been boosted as one of the major methods for studying PNDs.^6^ The default mode network (DMN) and DMN‐like network has been found across the species and the components were similar among rodents and mankind.^7^ Because of the fact that the DMN is responsible for cognitive function and mind wandering,^8^ the DMN could be used as a useful tool for studying disease mechanism, development and treatment effect. A great number of rs‐fMRI studies proved significant evidence for DMN changes in neurocognitive disorders,^9^, ^10^ however, one of the major problems for rs‐fMRI analysis is reproducibility.^11^ Although there had already been standard protocols for doing rs‐fMRI analysis,^12^ minor differences could still be found in different times of data analysis.^13^ Meanwhile, lack of methods' details and algorithm code undermines the interpretability.^14^ In this way, improving the reproducibility and interpretability behind DMN changes and data analysis are significant measures for improving the scientific value for rs‐fMRI studies and data analysis.

Neuroinflammation is one of the most commonly accepted mechanism for the development of POD.^15^ Recently, a great number of studies shown that microRNA (miRNA) plays pivotal roles in PNDs,^16^, ^17^ modulating down‐stream signaling pathways. Recent years, several studies demonstrated long‐lasting miRNA changes in animal models,^18^ suggesting a promising direction for interpretability of rs‐fMRI data analysis. However, whether miRNA changes contributed to DMN changes in anesthesia and surgery induced neurocognitive decline is rarely reported.

The present study was designed to investigate objective changes behind complicated DMN disruption in POD‐like behavior. POD‐like behavior was induced by tibial fracture fixation surgery in aged male rat under isoflurane inhalation because it is one of the most commonly applied surgery in elderly patients in clinical practice and it is also high‐risk surgery for POD occurrence.^19^ Inflammatory related DMN changes were drawn from secondary analysis of our previous rs‐fMRI data.^20^ Sequencing was applied to find out changes in the expression of miRNA and target genes and was later compared with results analyzed from rs‐fMRI. The results of our study will not only be able to improve the understanding for mechanisms of POD‐like behavior, but also improve the reproducibility and interpretability of rs‐fMRI data analysis.

## 2 METHODS

### 2.1 Animals

Aged male Wistar rats (19 months, *n* = 48 in total) were obtained from *Beijing SPF Animals Laboratory* and housed in a light/day circle of 12/12 h with food and water ad libitum.

Delirium‐like behavior in treatment group was induced by internal tibia fracture fixation (*n* = 32). Briefly, rats were anesthetized by isoflurane (7%–8% for induction and 2%–3% for maintenance). After loss of consciousness, the rat was kept warm at 37°C. After carefully shaving and disinfection, a 0.3–0.6 cm vertical incision was made near the right tibial tubercle followed by a 0.3 mm inner diameter steel needle insertion into the tibial tubercle along the longitudinal axis of the tibial medullary cavity. Subsequently, the tibia was cut by half. After suture and stop bleeding, 0.5% ropivacaine was subcutaneously injected to relieve the pain. The sham group (*n* = 16) was treated by shaving, vertical incision at the tibial tubercle, suturing and 0.5% ropivacaine local anesthesia without other surgical treatments.

Another cohort of rats were selected from our previous study.^20^ Briefly, male aged Wistar rats (19 months, *n* = 5 for doing rs‐fMRI data collection on a 7.0 T MRI scanner) were intraperitoneally injected with lipopolysaccharide (LPS, 2 mg/kg) for inducing long‐term neuroinflammation since the detailed mechanism of POD has been recognized as neuroinflammation. Detailed parameters for rs‐fMRI scanning, data collection and data analysis could be found in the same study.

### 2.2 Behavioral tests

A separate cohort of animals underwent behavioral tests to make sure that anesthesia and surgery induced delirium‐like behavior (*n* = 8 for each group). The behavioral tests were chosen because these tests were proved to be effective in animal experiments for delirium‐like behavior.^21^, ^22^ Both test protocols were adopted from previously published articles.^23^, ^24^ For both behavioral tests, a black rectangular arena in 100 × 100 × 40 cm size was placed in the center of a dimly light room with 24°C in temperature and 50% in humidity. A rectangular frame was built around the arena and a camera was suspended at the top to track the rat's trajectory with an animal tracking system (DigBehv, version 4.2.5.220725, Shanghai Jiliang Software Technology Co., Ltd., Shanghai, China) was used for data analyzing. After the behavior tests on the last time point (day 7), all the rats went euthanasia by deep isoflurane anesthesia.

#### 2.2.1 Novel object recognition (NOR) test

The NOR task was used in assessing non‐spatial object memory in rodents,^25^ one of a major aspect in delirium‐like behavior.^26^

The test was divided into three sessions. Before the start of each session, the arena was cleaned and sterilized thoroughly by 75% alcohol and will not be used until the fully evaporation. In the first session (10 min), rat was gently placed in the arena to let the animal explore freely for habituation. In the second session (10 min), two identical objects were placed at the second quadrant and fourth quadrant. Rat was gently placed into the arena facing the wall and to let it explore freely. In the third session (5 min), two different objects (in cube and cylinder for one is familiar and the other is novel) were placed at the opposite quadrant (the second and the fourth) and let it explore the arena freely. The time spent for exploring each object and the recognition index (RI) percentage were recorded.

The RI percentage could be expressed as: RI (%) = time spent studying the new object/(time spent studying the new object + time spent studying the familiar object) × 100%.

#### 2.2.2 Open field test (OFT)

The OFT task was performed to test the anxiety behavior in rats after anesthesia and surgery, for anxiety behavior is a significant character and associated with delirium.^27^

The test was started by placing the rat at the center of the arena and let it explore the arena freely for 10 min. The arena was then wiped with 75% alcohol to eliminate the previous olfactory cue of the previous rat. The distance walked and the average speed was recorded during the test and was recorded. After the end of test, the arena was cleaned and sterilized thoroughly.

### 2.3 Tissue collection

Another cohort of animals were prepared for tissue collection (*n* = 32). Before surgery (day 0 for sham group) and on day 3, day 7 and day 31 after surgery (for treatment group), rats were terminated under 8% isoflurane (Baxter Healthcare, USA) anesthesia. The brain was carefully harvested, and the blood was taken from the *vena cava*. The blood sample were centrifuged at 4°C for 1000 *g* and 15 min.

Both sides of hippocampus were carefully isolated by a chilled ice‐cold blade on frosted glass and quickly cooled by liquid nitrogen. All the surgical instruments were carefully pre‐processed by RNase (Promega, USA) agent to prevent RNA from degradation.

#### 2.3.1 Protein suspension

Both sides of the hippocampus were grinded into small pieces and homogenized by RIPA lysis buffer to (P0013C, Beyotime, Beijing, China) to reach the concentration of 20 mg tissue/200 μg buffer. Prior to homogenization, PMSF (100 mM, P0100‐1, Solarbio, Beijing, China) was added to reach the concentration at 1 mM/mL. After centrifugation (Eppendorf Centrifuge, 5810R, China) at the speed of 1000 *g*, 4°C, the suspension was collected and stored at −80°C for further use (*n* = 5 for sham group on day 0 and *n* = 5 for treatment group on each time point after anesthesia and surgery).

#### 2.3.2 RNA isolation and sequencing

Total RNA (*n* = 3 for each time point in treatment group on day 3, 7, and 31, while *n* = 3 for sham on day 0) was extracted from hippocampus with Trizol (Qiagen, Germany) and was purified by RNase Away Reagent (Invitrogen, Paisley, UK) according to the manufacturer's instructions. Total RNA was measured by Qubit 3.0 (ThermoFisher Scientific). Quality control was performed by Agilent 2100 Bioanalyzer and only RNA samples with integrity number (RIN) no <7 would be used in further analysis.

Small RNA sequencing libraries were constructed according to the Illumia TruSeq RNA Sample Preparation Protocol. Briefly, 3′ and 5′ terminals of RNA adaptors, which were designed to target the actual ends of small RNA molecules, were titrated to 1 mg of high‐quality total RNA.

Reverse transcription was used to generate cDNA libraries and RT‐PCR was performed to amplify and add unique index for each library. Small RNA libraries were pooled, and 50 bases were sequenced for each cDNA molecule with an Illumia Hiseq 2500 sequencer.

#### 2.3.3 Real‐time polymerase chain reaction (RT‐PCR)

The RT‐PCR was performed to re‐test the result of sequencing (*n* = 5 for each time point in treatment group on day 3, 7 and 31 while *n* = 5 for sham on day 0). Once the RNA was extracted from the hippocampus by Trizol agent (Invitrogen, Paisley, UK), total RNA was measured by NanoDrop 2000 (ThermoScientific, Waltham, MA, USA) and cDNA was constructed by the M‐MLV Reverse Transcription Kit according to the manufacturer's instructions. The cDNA amplification was performed by the ABI 7500 Fast Real‐Time PCR System (Applied Biosystems, Waltham, MA, USA) for 45 cycles (for each duration, 94°C for 2 min, annealing for 5 s at 94°C, for 30 s at 60°C and extension at 72°C for 10 min). Levels of RNA expression was calculated by SYBR Green direct method, with all the data analyzed by the 2^ΔΔ*C*t^ method by target gene expression in rat hippocampus by β‐actin expression. The sequences of primer are presented in Table 1.

**TABLE 1:** Primers in RT‐PCR.

	Forward Primers (5′→3′)	Reverse Primers (5′→3′)
miR‐34b‐5p	CGGTGCTCGGTTTGTAGGC	TTGATGGCAGTGGAGTTAGTGATT
*Tab2*	CAGCACCTCACAGACCC	TTGAAGCCGTTCCATCC
*Tubb3*	CCTTCATCGGCAACAGCA	GCCTCGGTGAACTCCATCT
miR‐328‐5p	TGGCTGAAGAACATGGGTGAG	CCTTGTGAGTTGGGCTGGA
*Gys1*	ATGCCGTCCTGTTTGGTT	TGGCGTGAGTGGTGAAGAT
*Nos1*	AGAGGAGGACGCTGGTGT	GGCGGTTGGTCACTTCA
*Pak6*	CCCACCCAAGCTAAAGAA	AGAGGCACCAGACACTCG
miR‐3505	AAGGGAAATACAAGACT	AATAAGACAGTCATGAAG
β‐Actin	TTTGAGGGTGCAGCGAACTT	ACAGCAACAGGGTGGTGGAC

#### 2.3.4 Enzyme‐linked immunosorbent assay (ELISA)

Cytokines including interleukin‐1 beta (IL‐1β, ab100768, Abcam, Cambridge, UK) and interleukin‐6 (IL‐6, ab234570, Abcam) were chosen because these cytokines had been proved to be able to be sensitive to neuroinflammation in aged rat brain.^28^, ^29^ All the cytokines were tested according to the manufacturers' instructions. Inflammatory cytokines in systemic level were tested by serum while in the hippocampus were tested by protein suspension (*n* = 5 for sham group on day 0 and *n* = 5 for treatment group on day 3, day 7 and day 31 for both serum and hippocampus).

### 2.4 Default mode network (DMN)

The rat DMN was defined by 12 regions of interest (ROI, 6 ROIs for each side of the brain), which were selected from the *Paxino & Watson* space.^30^, ^31^ All the ROIs could be found in the same digital mask.

The ROIs were defined as nodes and the functional connectivity (FC) was defined by the *Pearson* correlation coefficients (CCs) among two nodes calculated by Gretna.^32^ Weighted undirected 12 × 12 matrix was built for aged rats underwent LPS (represent as day 3, day 7 and day 31) and normal saline (represent as day 0). Because the hippocampus was used for sequencing and validation in our study, the hippocampus (left‐/right side) was used for seed‐region and the FC was calculated by the CCs between either L‐Hip or R‐Hip and other ROIs.

### 2.5 Sequencing data analysis

Sequencing mainly includes the following steps: (1) Removing the low‐reads and adaptor‐only reads. (2) Removing the 3′ prime adaptors part. (3) Reads length that ranges from 18 to 30 nt will be used for subsequent process.

Conserved miRNAs were identified by comparing reads of siRNA with known miRNA collected in the database (http://www.mirbase.org/) using Bowtie 1.1.1, with 1 mismatch allowed. Novel siRNA was identified from mismatch reads by miRDeep2 software. Only those with precursors found in the genome library were identified as conserved or novel miRNAs.

Potential miRNA targets were predicted using miRanda with default parameters. Different expression was analyzed using edgeR and limma package. Function annotation of target genes and enrichment analysis for the DEG of miRNA were performed with KOBA3.0.

### 2.6 Statistical analyses

Statistical data were analyzed by the IBM SPSS 26.0 (IBM, Chicago, IL, USA). Parametric data were presented as mean ± standard deviation (SD), while non‐parametric data were presented as median and interquartile range (IQR). After testing for normal distribution by *Shapiro–Wilk* test, data in ELISA and RT‐PCR were analyzed by one‐way ANOVA followed by *Bonferroni* or *LSD* post hoc analysis. Data in behavior tests were analyzed by two sample *t* test. Data in rs‐fMRI and sequencing were analyzed during data processing. The value of *p* < 0.05 was considered statistically significant after *Bonferroni* or *LSD* post‐hoc analysis.

## 3 RESULTS

### 3.1 Delirium‐like behavior was observed in aged rat after anesthesia and surgery

**FIGURE 1:**
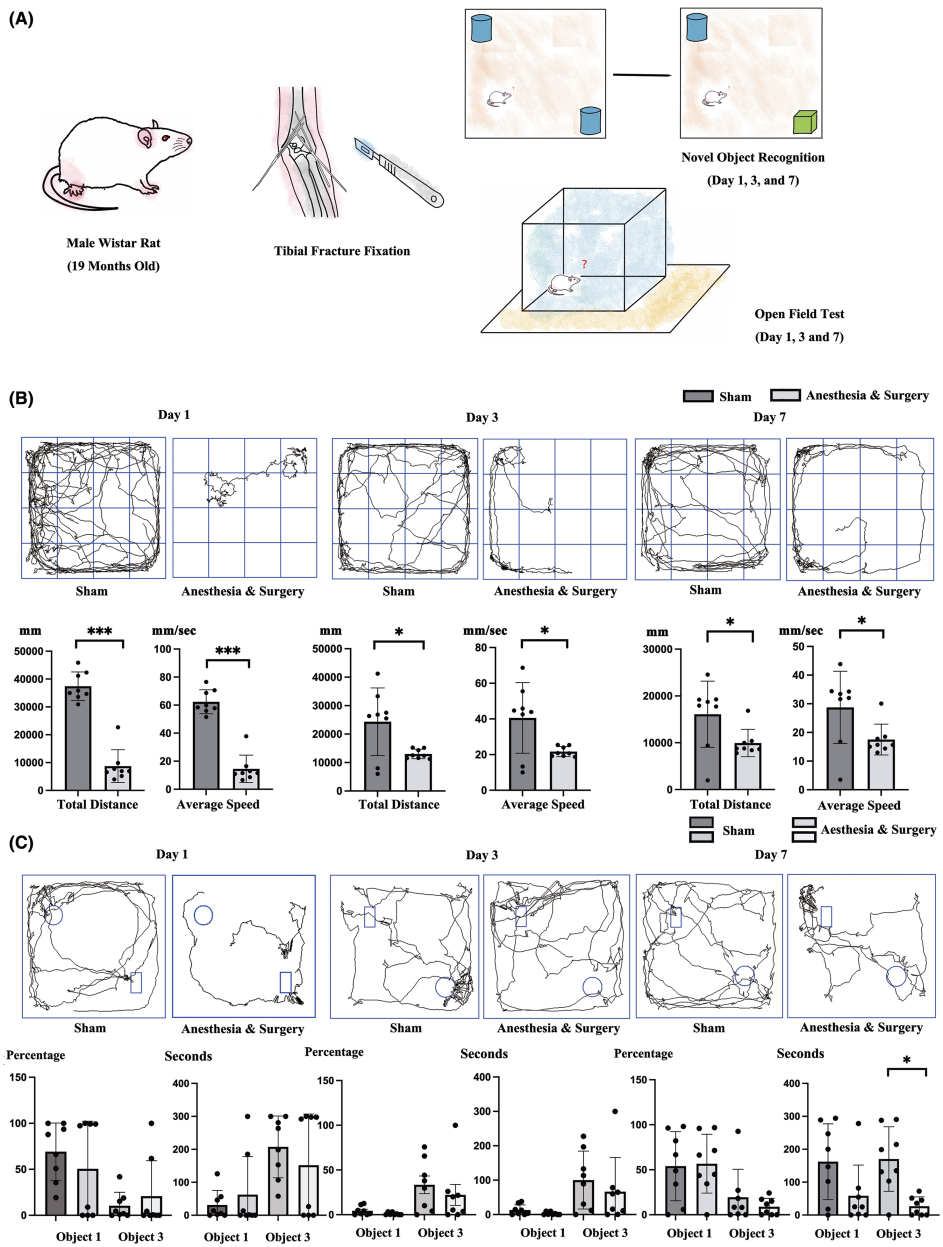
Anesthesia and surgery induced delirium‐like behavior. (A) Rats under isoflurane anesthesia and surgery underwent NOR and OFT on day 1, day 3 and day 7 after treatment. (B) OFT showed significantly decreased total distance and average speed in rats underwent anesthesia and surgery. (C) NOR showed decreased recognition index (RI) and time spent on day 3 and significantly decreased time spent on day 7 in rats underwent anesthesia and surgery. Data were expressed as mean ± standard deviation (SD). *p < 0.05, ***p < 0.001.

New object recognition (NOR) and OFT were adopted to test POD‐like behavior in rats (Figure 1A).

For OFT, the distance walked, and the average speed was recorded (Figure 1B). Significant decreases in walking distance were observed in rats undergoing anesthesia and surgery on day 1, day 3 and day 7 as compared with rats in the sham group (37,446.4850 mm vs. 8739.1475 mm, 24,346.5425 mm vs. 13,017.2038 mm, and 16,096.2663 vs. 9947.2075 mm, *p* < 0.001, *p* = 0.031, and *p* = 0.039, Figure 1B). For average speed, anesthesia and surgery also induced significant differences on day 1, day 3 and day 7 (62.4150 mm/s vs. 14.5650 mm, 40.5788 mm/s vs. 21.6950 mm/s, and 28.7413 mm/s vs. 17.5175 mm/s, *p* < 0.001, *p* = 0.031, and *p* = 0.036).

For NOR, the time spent for exploring each object and the recognition index (RI) percentage were recorded (Figure 1C). One day after anesthesia and surgery, no significant decrease in time spent on novel object and recognition index (RI) was found. On day 3, significant longer time spent in sham group during the training process was observed (12.4238 s vs. 3.0050 s for object 1 and 283.9175 s vs. 69.8662 s for object 2, *p* = 0.032 and 0.035, independent *t* test) with no statistically difference in RI, although a slightly longer RI for object 3 in the sham group was still observed (33.4113% vs. 22.1263%, *p* = 0.474 for object 3, independent *t* test). On day 7 after anesthesia and surgery, significant differences in time spent on novel object (2326.7262 s vs. 11.6875 s, *p* = 0.026 for object 3) was observed although no statistical difference in RI was found (54.0113% vs. 55.3581%, *p* = 0.882 for object 3).

### 3.2 Long‐term neuroinflammations were found in POD rats

**FIGURE 2:**
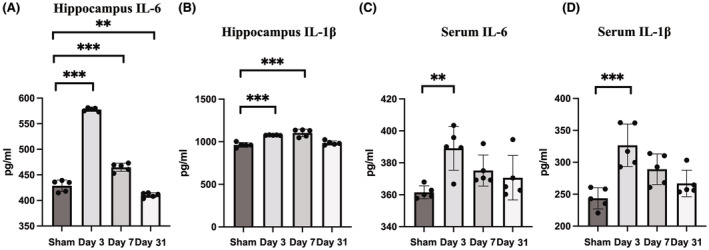
Inflammatory responses in rat. (A, B) Inflammatory responses in aged rat hippocampus. (C, D) Inflammatory responses in aged rat serum. Data were expressed as mean ± standard deviation (SD). **p < 0.01, ***p < 0.001.

We both tested inflammatory cytokines in the acute (within 7 days) and chronic phase (after 31 days) in the rat brain to see if POD induced both short‐term and long‐term neuroinflammation (Figure 2).

POD‐like behavior induced significantly changed IL‐6 level (one‐way ANOVA, *F* = 500.846, *p* < 0.001) on day 3, day 7 and day 31 (*p* < 0.001 for day 3, day 7 and *p* = 0.009 for day 31, *Bonferroni* post hoc analysis, compared with sham group). For the level of IL‐1β, significantly variation was also observed (one‐way ANOVA, *F* = 28.718, *p* < 0.001) on day 3 and day 7 (*p* < 0.001 for day 3 and day 7, *Bonferroni* post hoc analysis, compared with sham group). On day 31, a slightly increase was still observed while no significant difference was observed (*p* = 1.00).

Systemic level of IL‐6 and IL‐1β were observed for significantly variations (one‐way ANOVA, *F* = 5.358, *p* = 0.010 for IL‐6 and *F* = 10.47, *p* < 0.001 for IL‐1β) on day 3 (*p* = 0.007 for IL‐6 and *p* < 0.001 for IL‐1β, *Bonferroni* post hoc analysis as compared with sham group).

### 3.3 Long‐term RNA changes were found in POD rats

**FIGURE 3:**
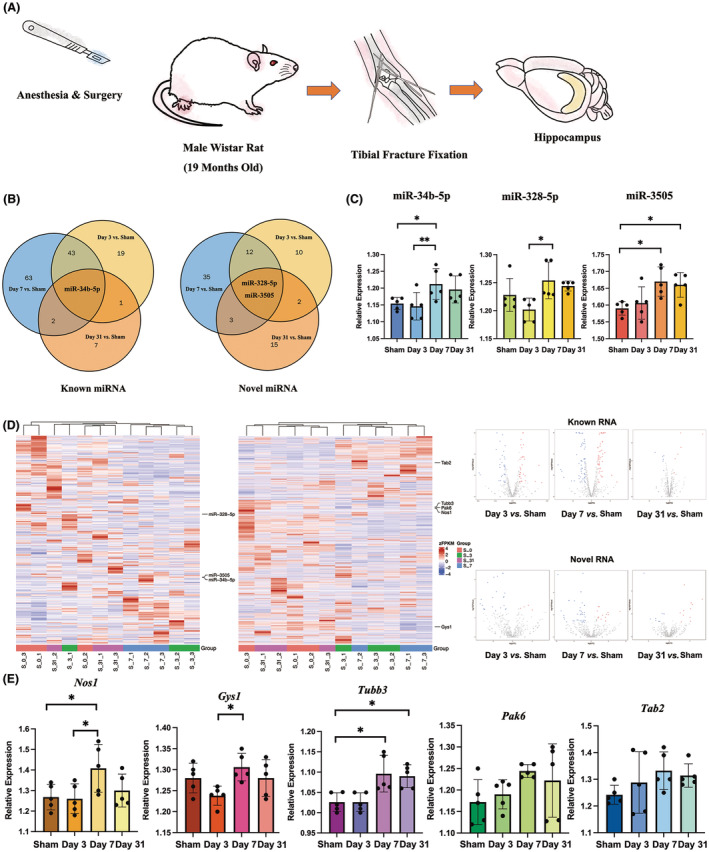
Sequencing results in aged rat hippocampus. (A) Hippocampus was taken from rats in both treatment and sham group. (B) Persistent miRNA changes were found in both short‐term (<7 days) and long‐term (>30 days). (C) Validation results showed significantly changed miR‐34b‐5p, miR‐328‐5p and miR‐3505 after anesthesia and surgery. (D) Expression changes in miRNA and gene in hippocampus by sequencing. (E) Genes including *Nos1*, *Gys1* and *Tubb3* were shown changed after anesthesia and surgery in hippocampus. Data were expressed as mean ± standard deviation (SD). *p < 0.05, **p < 0.01.

Sequencing was applied in both sides of hippocampus. Following sequencing, persistent changes including miR‐34b‐5p, miR‐328‐5p and miR‐3505 were found in rat hippocampus (Figure 3A,B). Afterwards, RT‐PCR was applied for further validation.

For further analysis and validation (Figure 3C,D), POD‐like behavior induced significant changes in miR‐34b‐5p (*p* = 0.027, *F* = 4.002, one‐way ANOVA). Further analysis and validation revealed significant increase on day 7 (*p* = 0.02, *LSD* post hoc analysis). For miR‐328‐5p, significant changes were also observed on day 7 (*p* = 0.030, *F* = 3.852, one way ANOVA and *p* = 0.039, *Bonferroni* post hoc analysis as compared with day 3). For miR‐3505, significant changes were observed (*p* = 0.007, *F* = 5.711, one‐way ANOVA) as compared with rats without surgery on day 7 and day 31 (*p* = 0.023 and *p* = 0.042, *Bonferroni* post hoc analysis).

We further detected the target gene expression by the *KEGG* database (Figure 3D). When referring to *KEGG* terms, genes involved in cognitive change and neuroinflammation were identified. For miR‐34b‐5p, *Tab2* (TNFα signaling pathway) and *Tubb3* (gap junction) were identified. For miR‐328‐5p, *Gys1* (AMPK signaling pathway), *Nos1* (Alzheimer's disease) and *Pak6* (ErbB signaling pathway) were identified (Figure 3). For miR‐3505, no cognitive related genes were found.

To examine if we got the correct result, RT‐PCR was performed to validate the above results (Figure 3E). POD‐like behavior induced significant differences in the expression of *Gys1* (*p* = 0.039, *F* = 3.532, one‐way ANOVA), *Nos1* (*p* = 0.049, *F* = 3.253, one‐way ANOVA) and *Tubb3* (*p* = 0.002, *F* = 8.149, one‐way ANOVA). Further analysis showed the expression of *Gys1* significantly changed on day 7 (*p* = 0.034, *Bonferroni* post hoc analysis as compared with day 3), *Nos1* showed significant differences on day 3 and day 7 while the expression of *Tubb3* showed significant increase on day 7 and day 31 (*p* = 0.019 and 0.014, *LSD* post hoc analysis for *Nos 1* and *p* = 0.013 and 0.031, *Bonferroni* post hoc analysis for *Tubb3*, as compared with sham group). No significant differences were observed in other genes (*p* = 0.164 for *Pak6* and *p* = 0.282 for *Tab*2, one‐way ANOVA).

### 3.4 Significant correlation between the brain network change and microRNA and the gene expression

**FIGURE 4:**
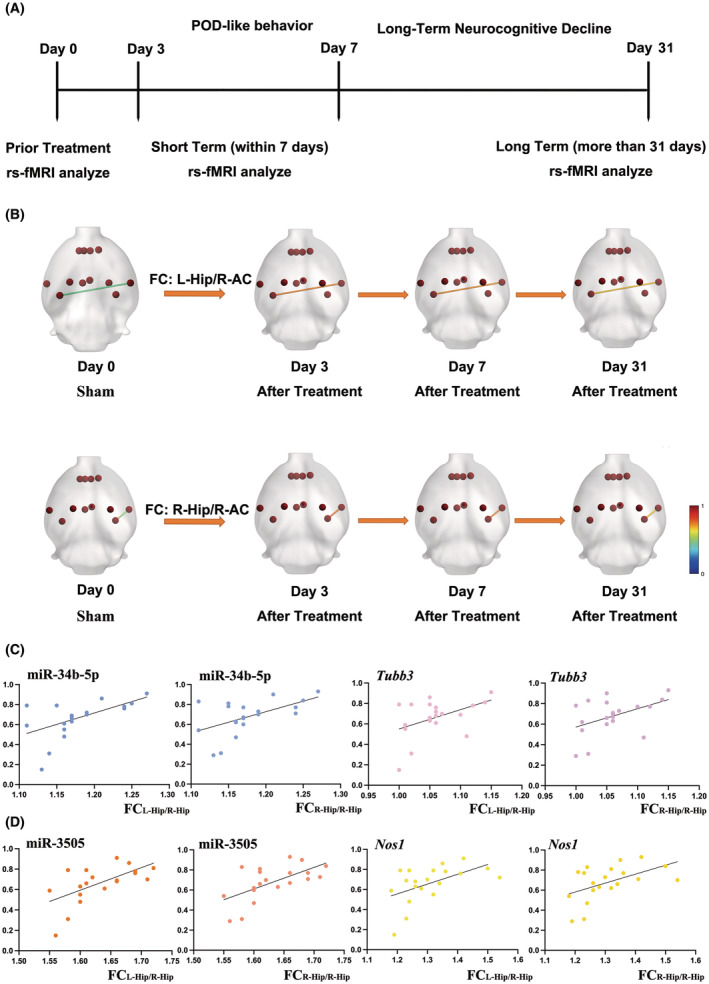
Correlations for inflammatory expressions and default mode network (DMN) disruption. (A) Time points for studying DMN. (B) Significant FC changes in DMN using hippocampus as seed region. (C) Significant correlations were found in the expression of miR‐34b‐5p and *Tubb3* with FC changes in DMN. (D) Significant correlations were found in the expression of miR‐3505 and *Nos1* with FC changes in DMN.

We further used aged rat to identify brain network change induced by long‐term neuroinflammation. By re‐analyzing our previous data using the hippocampus as seed region, we found persistent changes including FC_L‐Hip/R‐AC_ (*p* = 0.017, *F* = 4.566, one way ANOVA) and FC_R‐Hip/R‐AC_ (*p* = 0.026, *F* = 4.023, one‐way ANOVA) (Figure 4A,B).

By using the *Pearson* correlation, significant correlations were found in miR‐34b‐5p (*p* = 0.012, *r* = 0.548 for FC_L‐Hip/R‐AC_ and *p* = 0.012, *r* = 0.548 for FC_R‐Hip/R‐AC_) and miR‐3505 (*p* = 0.006, *r* = 0.592 for FC_L‐Hip/R‐AC_ and *p* = 0.005, *r* = 0.607 for FC_R‐Hip/R‐AC_). However, no significant correlations were found for miR‐328‐5p (*p* = 0.138 for FC_L‐Hip/R‐AC_ and *p* = 0.100 for FC_R‐Hip/R‐AC_).

We next analyzed the correlations between target genes and FC changes. Since the miR‐34b‐5p and the *Tubb3* both had significant changes, it was first analyzed by *Pearson* correlation with brain network changes. Moderate correlations were found in *Tubb3* with FC_L‐Hip/R‐AC_ (*p* = 0.043, *r* = 0.456) and with FC_R‐Hip/R‐AC_ (*p* = 0.044, *r* = 0.455). Then, genes that had significant changes after anesthesia and surgery were also analyzed although the up‐stream miRNA was not significantly correlated with brain network changes. Significant correlations were observed in *Nos1* with FC_L‐Hip/R‐AC_ (*p* = 0.020, *r* = 0.517) and with FC_R‐Hip/R‐AC_ (*p* = 0.025, *r* = 0.0499). For *Gys1*, no significant differences were found in both FC_L‐Hip/R‐AC_ and FC_R‐Hip/R‐AC_ (*p* = 0.375 and *p* = 0.312).

Specifically, we also analyzed the correlations between FC changes in genes that were not significantly changed in PCR verification to check if these genes also contributed to brain network changes. No significant correlations were found for *Tab2* (*p* = 0.162 for FC_L‐Hip/R‐AC_ and *p* = 0.140 for FC_R‐Hip/R‐AC_) and *Pak6* (*p* = 0.256 for FC_L‐Hip/R‐AC_ and *p* = 0.299 for FC_R‐Hip/R‐AC_).

## 4 DISCUSSION

Our result demonstrated a significant correlation between the expression of microRNA, target gene and DMN disruption in anesthesia and surgery induced POD‐like behavior, which revealed a potential direction for improving interpretability and reproducibility in rs‐fMRI data analysis.

In patients with delirium, rs‐fMRI revealed significant FC changes and selected several significant brain regions for long‐term cognitive decline. Among the studies, one of the most significant character is DMN changes.^33^, ^34^ In our study, DMN changes were obtained from neuroinflammation model instead of POD model, which was based on thoroughly consideration. One of the main mechanisms for POD is neuroinflammation^15^, ^35^ while both LPS exposure and bone fracture fixation surgery have all been used for inducing delirium‐like behavior in neuroscience research.^36^, ^37^, ^38^, ^39^ These results provided evidence for the rational use of neuroinflammation model in POD research. When comparing rs‐fMRI results obtained from neuroinflammation model, POD model and even POD patients, results including significant decrease in orbito‐cortex and hippocampus related FC,^33^ decrease in DMN connectivity,^40^ reduced connectivity between prelimbic frontal cortex and other brain regions,^41^ and disconnections between the lower subcortical regions^34^ were all observed, suggesting that the results are comparable in two models. Furthermore, influence of pain on surgery fixation induced POD model is hard to avoid and whether the network change was resulted from pain or cognitive decline is difficult to determine.^42^, ^43^ In this case, mixed results may reduce the quality of the study. When using rats as models for studying neuroinflammation, the only persistent DMN changes that could survive the strict *Bonferroni* post hoc analysis throughout the short‐ and long‐term is the FC between Hip and R‐AC. Although the Hip is a traditionally used region for studying cognitive dysfunction, the R‐AC is a rather seldom used region in PNDs. These differences call for increasing the reproducibility and interpretability for rs‐fMRI data analysis.

In our study, after confirming by NOR and OFT for POD‐like behavior, including miR‐34b‐5p, miR‐328‐5p and miR‐3505 were found to have effective long‐term changes. Although POD has been traditionally recognized as a transient but benign process, for which the symptom only lasts for a short of time (mostly within 7 days after anesthesia and surgery),^44^ our results suggested that POD‐like behavior induced long‐term RNA expression changes (for up to 31 days) in aged rat hippocampus behind a rather normal cognitive function (as what the behavior tests indicated). This is also consistent with clinical findings that delirium is highly correlated with long‐term cognitive decline.^45^

One of the most significant reasons for neuroinflammation induced by surgery is the broke up of blood brain barrier (BBB).^35^ Our study revealed the circuit of miR‐34b‐5p/*Tubb3* and a significant long‐term increase in *Tubb3* expression after anesthesia and surgery. By referring to the *KEGG* database, the change in the expression of *Tubb3* revealed a long‐term change for the permeability of BBB and gap junction. This can also explain the reason for persistent neuroinflammation after POD‐like behavior in aged rat. Another significant finding is that the circuit of miR‐328‐5p/*Nos1* may participated in POD‐like behavior induced long‐term cognitive decline, while it has to be noted that only *Nos1* but not miR‐328‐5p is significantly correlated with Hip related FC. The miR‐328‐5p is a newly found microRNA in POD‐like behavior which is rarely seen in traditionally built *KEGG* database. The *Nos1* gene is of great significance in AD development according to the *KEGG* database. For example, the repeat of *Nos1* promoter participated in interaction with APOE4 in AD pathogenesis.^46^ Whether targeting miR‐34b‐5p/*Tubb3* or miR‐328‐5p/*Nos1* has a significant effect on restoring long‐term cognitive function still need further investigation.

Neuroinflammation induced brain network change has been used as a sensitive marker for studying mechanisms of disease development and cognitive decline. The present study further correlated the brain network changes with micro‐RNA and gene expression after anesthesia and surgery in order to find out whether these changes contributed to brain network changes. In our study, the expression of miR‐34b‐5p, *Nos1* and *Tubb3* all have significant correlations in FC_L‐Hip/R‐AC_ and FC_R‐Hip/R‐AC_ while no other significant correlations were found in DMN. The result confirmed our hypothesis that the brain network changes is not simply results shown by analyzing rs‐fMRI data but are significant biological changes in brain. The changes in miRNA and target genes all showed significant contributions for DMN disruption.

Recently, the auditory‐hippocampus has been characterized a significant pathway for cognitive function. In rodent animals, the projection runs from entorhinal cortex (ERC), by the trisynaptic loop, through the DG, CA3 and CA1 region from where the output is routed back to neocortex.^47^ The hippocampus not only binds across sensory modalities but situates these multimodal objects in a spatiotemporal context to form memories of particular episodes.^48^ In human subjects, the hippocampus BOLD activity was reported to be significantly increased when retrieve specific autobiographical episodes while in rodent animals this is hard to be tested. Considering the difference between rodent animals and human beings in cortex, whether the FC between hippocampus and auditory cortex reveals new pathway for long‐term cognitive decline after POD‐like behavior needs further confirmation.

The present study has several limitations. Firstly, the rs‐fMRI data was acquired from long‐term neuroinflammation model. The reasons for doing this is that pain caused by anesthesia and surgery may also influence the DMN and thus this model without surgery was adopted. Secondly, we didn't test the effects of miR‐328‐5p/*Nos1* and miR‐34b‐5p/*Tubb3* on rat central nerve system because the aim of the present study is to improve the interpretability and reproducibility behind rs‐fMRI signal changes. Thirdly, sequencing results were drawn from rats instead of mankind. Our results should be further validated in human subjects for better understanding of the POD mechanisms.

## 5 CONCLUSION

Our results demonstrated that short‐term POD‐like behavior resulted in long‐term microRNA abnormal expression, neuroinflammation in hippocampus while POD‐like behavior resulted long‐term change of miR‐34b‐5p/*Tubb3* may be responsible for long‐term changes in hippocampus related DMN network FC in long‐term neuroinflammation. The result increased reproducibility and interpretability for standardized rs‐fMRI data analysis, as well as providing potential targets for postoperative delirium treatment.

## FUNDING INFORMATION

This work was supported by grants from Capital's Funds for Health Improvement and Research (ID. CFH2024‐4‐20114), Training Fund for Open Projects at Clinical Institutes and Departments of Capital Medical University (ID. CCMU2023ZKYXY006) and Post‐subsidy funds for National Clinical Research Center Ministry of Science and Technology of China (ID. 303–01–001‐0272‐03).

## CONFLICT OF INTEREST STATEMENT

The authors declare that there are no conflicts of interest.

## Data Availability

The data that supports the findings of this study are available from the corresponding author upon reasonable request.
